# Stereophotogrammetric head shape assessment in neonates is feasible and can identify distinct differences between term-born and very preterm infants at term equivalent age

**DOI:** 10.1038/s41598-021-00680-1

**Published:** 2021-10-27

**Authors:** Petra Santander, Anja Quast, Johanna Hubbert, Laura Juenemann, Sebastian Horn, Kai O. Hensel, Philipp Meyer-Marcotty, Jana-Katharina Dieks

**Affiliations:** 1 Department of Orthodontics, University Medical Center, Robert-Koch-Straße 40, 37075 Göttingen, Germany; 2 Department of Pediatric Cardiology, Neonatology and Intensive Care Medicine, University Medical Center, Georg-August University Göttingen, Robert-Koch-Straße 40, 37075 Göttingen, Germany

**Keywords:** Paediatric research, Diseases

## Abstract

The development of head shape and volume may reflect neurodevelopmental outcome and therefore is of paramount importance in neonatal care. Here, we compare head morphology in 25 very preterm infants with a birth weight of below 1500 g and / or a gestational age (GA) before 32 completed weeks to 25 term infants with a GA of 37–42 weeks at term equivalent age (TEA) and identify possible risk factors for non-synostotic head shape deformities. For three-dimensional head assessments, a portable stereophotogrammetric device was used. The most common and distinct head shape deformity in preterm infants was dolichocephaly. Severity of dolichocephaly correlated with GA and body weight at TEA but not with other factors such as neonatal morbidity, sex or total duration of respiratory support. Head circumference (HC) and cranial volume (CV) were not significantly different between the preterm and term infant group. Digitally measured HC and the CV significantly correlated even in infants with head shape deformities. Our study shows that stereophotogrammetric head assessment is feasible in all preterm and term infants and provides valuable information on volumetry and comprehensive head shape characteristics. In a small sample of preterm infants, body weight at TEA was identified as a specific risk factor for the development of dolichocephaly.

## Introduction

Long-term neurodevelopmental outcome is a key determinant for quality of neonatal care. Neurodevelopment is particularly not only affected by intracerebral lesions such as leucomalacia or hemorrhage but also by brain growth.

Head circumference (HC) has been established as a marker reflecting brain development^1^, which has been utilized as a clinical endpoint in contemporary trials investigating nutritional support in very preterm infants^2^. However, measuring the outer circumference of a newborn’s head is a rather crude two-dimensional (2D) surrogate parameter to assess brain volume.

Symmetry of a head’s morphology is presumably a more comprehensive measure reflecting head development during early life. Both symmetrical and asymmetrical non-synostotic head shape deformities do not only represent a cosmetic problem but may also cause functional impairment such as developmental delay^3^. For deformational plagiocephaly and brachycephaly for example, an—not necessarily causal—association between the degree of deformity and poorer neurocognitive performance has been shown. This suggests that assessment of head shape deformity may help to identify children at risk for developmental delay^3^. Further issues include asymmetry of the temporomandibular complex with ear shifting, facial asymmetry and malocclusion, the latter being a risk factor for dental caries, traumatic dental injuries and problems of the temporomandibular joint^4–6^. Notably, there is no evidence that HC is a trustworthy parameter in infants with head shape deformities.

Recent research projects in both preterm and healthy term infants shed a light on important aspects such as the detection of risk factors, standardized classification, prevalence and assessment tools for head shape deformities^7^. Known risk factors include pre-, peri- and postnatal parameters as well as male sex, multiple birth, and being a firstborn infant^8^. Moreover, preterm infants have been shown to feature a particular risk for the development of microcephaly^9^ and head shape deformities during the first weeks of life^10,11^ and infancy^12^. These findings have been associated with an impaired neurodevelopmental outcome^13^.

Currently, head shape quantification is still not standardized, and deformities are often only assessed by visual impression or simple—but non-comprehensive—manual cephalometric methods such as calipers. Reference values for non-synostotic symmetrical and asymmetrical head shape deformities have been proposed^14^. To overcome this gap, we recently reported the feasibility of a portable and non-invasive stereophotogrammetric head shape assessment device in preterm infants that serves as a translation of this imaging technology from orthodontics into neonatal care^15^. Before our report, to the best of our knowledge, stereophotogrammetry has been used in head assessment in infants from four months of age^16^. As the present knowledge is still very limited in newborns and infants, our study aims are to present data on head shape and volume analyses obtained by stereophotogrammetry in very preterm infants compared to term infants at term equivalent (TEA) age.

## Patients and methods

### Patients

As part of a longitudinal observational study, 25 very preterm infants and 25 sex-matched term infants underwent stereophotogrammetric three-dimensional (3D) head imaging at TEA. Written informed parental consent was obtained prior to study image acquisition. The study had been approved by the ethics committee of the Georg-August-University medical centre (ethics proposal 19/2/18) and was performed in accordance with the amended Declaration of Helsinki.

Inclusion criteria into the study group were birth weight of below 1500 g and / or gestational age (GA) below 32 completed weeks. Patients of the control group had any body weight and a GA of 37–42 weeks.

Exclusion criteria for all patients were palliative care or any critical / deteriorating clinical health condition. For the healthy term control group, syndromal appearance or confirmed disease, clinically apparent cephalic or (sub-) galeal hematoma, other head / scalp lesions and any condition with an impact on head shape or neurological development were defined as additional exclusion criteria. Syndromal appearance or disease and birth trauma were assessed by at least one board certified neonatologist.

In preterm infants, 3D images were acquired at corrected TEA (between 37 and 42 weeks) and in term infants during the first week of life. The following data were obtained for each infant: Sex, multiple birth, GA, body weight (including percentiles) and HC at birth (measured one time to resemble the regular clinical day-to-day routine). Small for gestational age (SGA) was defined as body weight < 3rd percentile^17^. Analysed perinatal characteristics included birth presentation, mode of delivery and presence of asphyxia. At the time of image acquisition, postmenstrual age, body weight (including percentiles) and HC were recorded. Neonatal morbidity was determined by the presence of bronchopulmonary dysplasia (BPD), necrotizing enterocolitis (NEC), intracerebral hemorrhage (ICH) III-IV°, culture-proven or clinically determined sepsis^18^, retinopathy of prematurity (ROP) III-IV°, periventricular leucomalacia (PVL) and respiratory support. Respiratory support was subclassified into total duration of respiratory support, days of nasal continuous positive airway pressure, days of nasal high-flow therapy and days of invasive mechanical ventilation. Independent of the applied mode of respiratory support, all patients were equally placed in their incubators in prone and supine positions.

### Image acquisition and reconstruction

**Figure 1:**
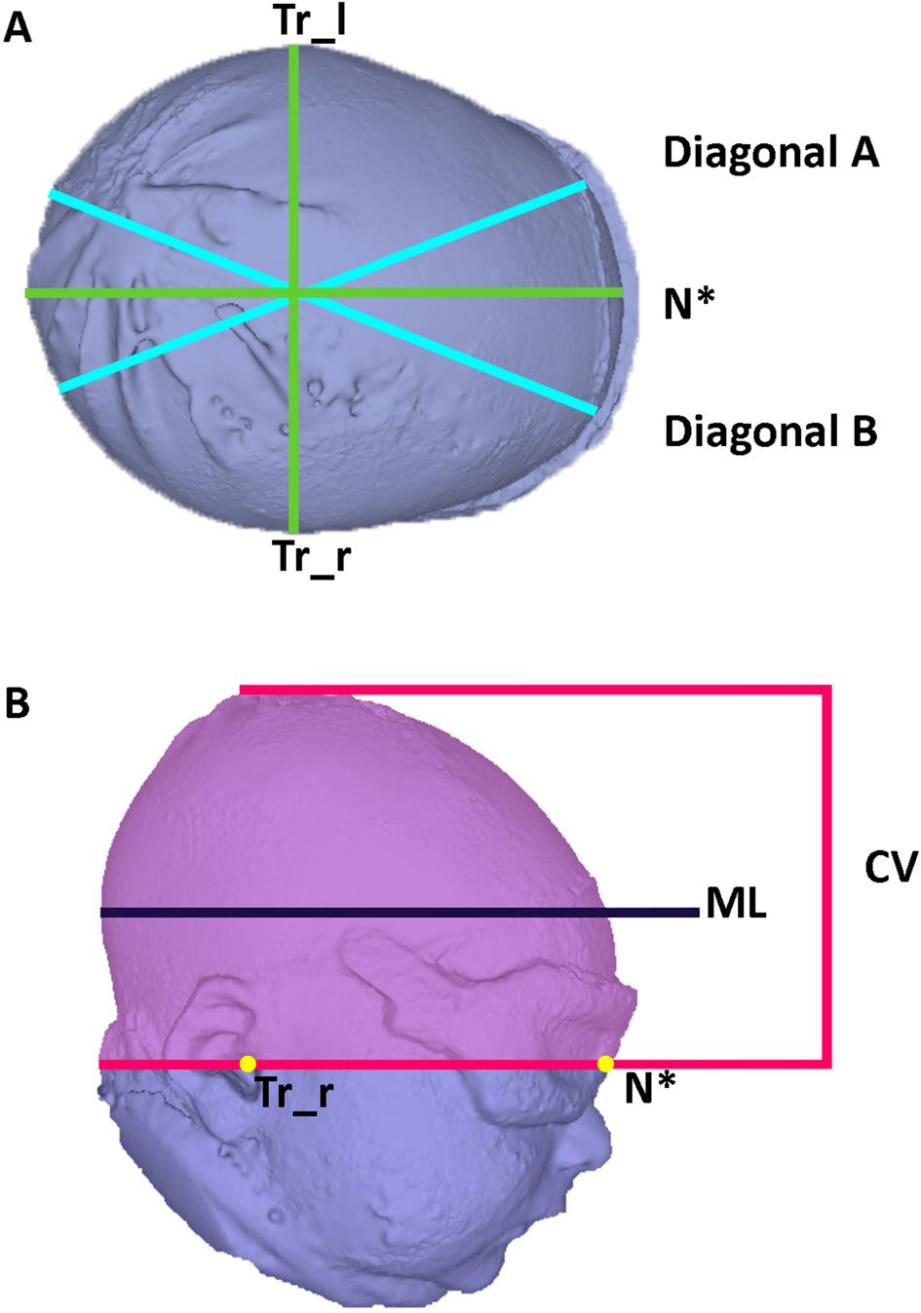
Clinical setting of image acquisition (**A**) during routine infant care and (**B**) while being held in the mother’s arms.

For image acquisition, a portable stereophotogrammetry Vectra H1 camera (Canfield Scientific, New Jersey, USA) was used. As previously described in detail, a 3D head imaging protocol with ten images from various angles was applied^15^. During the procedure, the infant’s head was covered with a soft nylon cap that contains textured applications to reduce hair artifacts and to facilitate subsequent 3D image reconstruction. To protect the infant’s eyes from potential irritation due to camera flashlight, a neonatal phototherapy eye protector (Neoshades®; Kreienbaum Neoscience GmbH, Langenfeld, Rheinland, Germany) was used in all infants. All image acquisitions were performed on the neonatal ward during patient care while the infants were placed in their incubator or bed or while being held by one of their legal guardians (Fig. 1).

For the 3D image reconstruction, VECTRA Analysis Module (VAM) software version 6.2.3 (Canfield Scientific, New Jersey, USA) and the MeshLab software version 2016.12 (Visual Computing Lab, ISTI-CNR, Pisa, Italy) were used.

### Measurements and definitions

**Figure 2:**
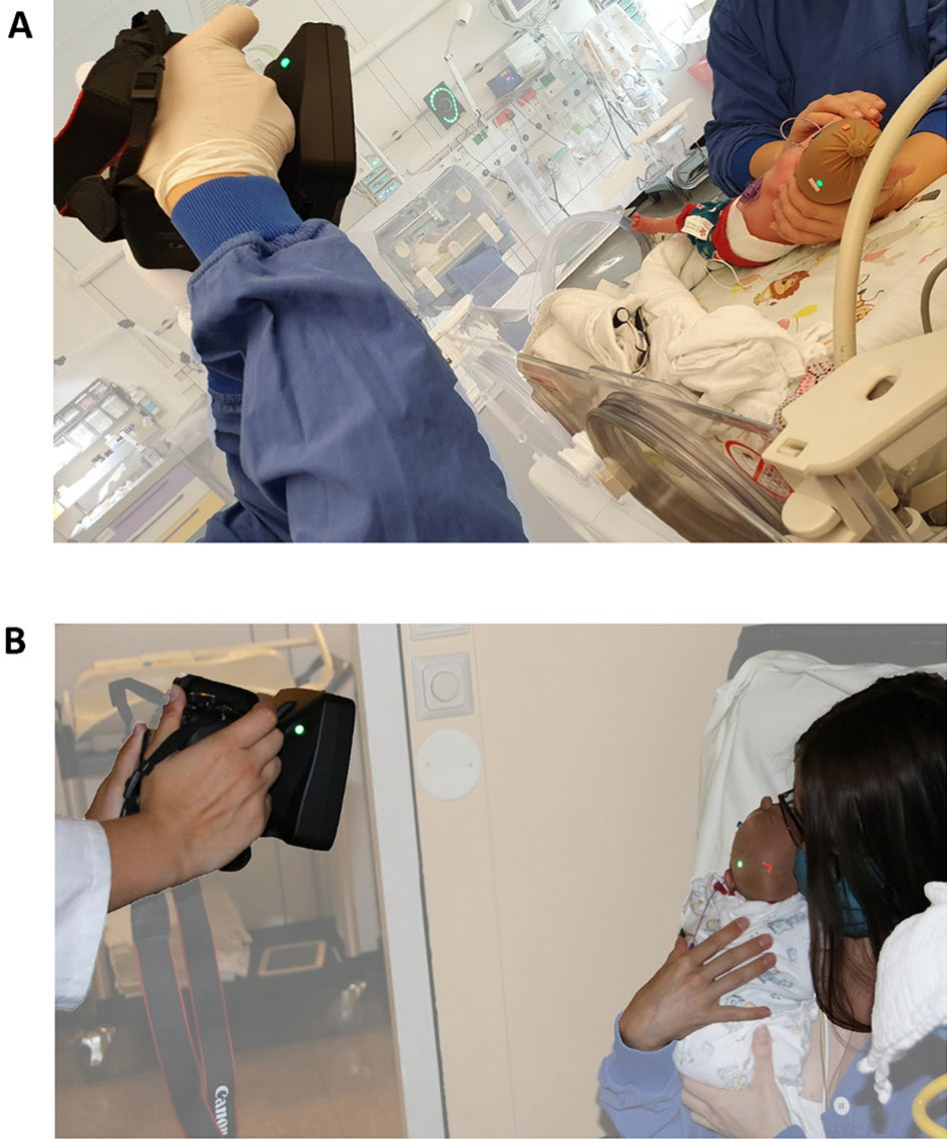
Display of the parameters for measurements of the infants’ heads. (**A**) Transversal view and (**B**) side view. *CV* cranial volume; *ML* measurement level; N* nasion*; *Tr_l/r* tragus left/right.

Digital measurements of the reconstructed 3D head model were performed using the Cranioform software version 4.0 (Cranioform, Alpenach Switzerland). Technical details and definitions are displayed in Table 1 and Fig. 2. HC was manually measured at the time of image acquisition.

**Table 1:** Definitions and descriptions of parameters and measurements. Since all infants wore an eye protection during image acquisition, the point nasion was located centrally between the eyes and referred to as nasion*.

Definition	Description
Measurement level (ML)	Head level with the major anterior–posterior expansion of the head
Head circumference (HC; cm)	Head perimeter around the ML
Cranial volume (CV; m^3^)	Total head volume cranial to the sagittal plane
Cranial index (CI; %)	Proportion of head width to length at ML Formula: $CI=\frac{head~widht}{head~lenght}\times 100$
Cranial vault asymmetry index (CVAI; %)	Difference between the longest (diagonal A) and shortest 30° diagonal (diagonal B) diameter radiating from the midsagittal plane at ML Formula: $CVAI=\frac{diagonal~A - diagonal~B}{major~diagonal}\times 100$

Non-synostotic head shape deformities were classified based on the described normative percentiles for cranial index (CI) and cranial vault index (CVAI) and analysed in light of the previously published reference values^14,19^. For this purpose, we applied mean reference values for female and male infants at zero to three months of age.

The CI was used to define symmetrical head shape deformities. Brachycephaly (i.e. increased head width related to head length) was classified as either mild (CI = 75th to 90th percentile; 85.6 to 89.5%), moderate (CI =  > 90th to 97th percentile; > 89.5 to 94.2%), or severe (CI > 97th percentile; CI > 94.2%), while dolichocephaly (i.e. increased head length in relation to head width) was classified as mild (CI = 10th to 25th percentile; 74 to 77.5%), moderate (CI = 3rd to < 10th percentile; 70.3 to < 74%), or severe (CI < 3rd percentile; CI < 70.3%).

Finally, asymmetrical head shape deformity plagiocephaly (i.e. diagonal asymmetry of the head) was classified using the CVAI as mild (CVAI = 75th to 90th percentile; 3.7 to 5.3%), moderate (CVAI =  > 90rd to 97th percentile; > 5.3 to 7%), or severe (CVAI > 97th percentile; > 7%).

### Biostatistical analyses

Patient data was handled using Microsoft Excel version 1906 (Redmond, WA USA). Numerical variables are expressed as median (interquartile range), categorical variables as percentages. Statistical analyses were conducted using SPSS Statistics version 26 (IBM, New York, USA). All data were assumed as non-normally distributed. Comparisons between the study and control group were performed using the Mann–Whitney-U-Test for independent samples. Correlations were assessed by Spearman's rank correlation coefficient. Relationship between head shape deformities and risk factors was analysed by Fisher’s exact test and multiple hierarchical regression. Finally, association between head shape and risk factors was analysed using multiple hierarchical regression. For all statistical tests, the level of significance was set to 0.05.

## Results

### Patients’ characteristics

Clinical characteristics of the preterm and the term infant group and data at time of image acquisition are displayed in Table 2.

**Table 2:** Clinical characteristics of the study sample.

		Preterm infants	Term infants
Number of patients		25	25
At birth	Gestational age (weeks)	30 (25.1–31.9; 2.7)	39.7 (37.3–41.3; 1.5)
	Sex (female / male)	9/16	9/16
	Multiple birth (%)	24	0
	Body weight (g)	1315 (550–1930; 573)	3605 (2190–4010; 698)
	Head circumference* (cm)	28.2 (22–32; 2.6)	35 (32–37.5; 1.5)
	Cesarean section (%)	92	32
	Cephalic birth presentation (%)	76	96
	Asphyxia (%)	0	0
At the time of image acquisition	Postmenstrual age (weeks)	40 (38.6–41.6; 1.7)	39.7 (37.3–41.3; 1.5)
	Body weight (g)	3480 (1900–4750; 1045)	3605 (2190–4010; 698)
	Head circumference* (cm)	36 (30–38.5; 2.8)	35 (32–37.5; 1.5)

*Manually measured; numerical data are displayed as median (minimum–maximum; interquartile range).

### Relationship between head circumference, cranial volume and body weight

A statistically significant correlation between the digitally measured HC and the CV in both study groups (preterm infants r = 0.950, *p* < 0.001; term infants r = 0.797, *p* < 0.001) was found. Further, even in moderately to severely plagiocephalic and dolichocephalic preterm and term infants, digitally measured HC and CV exhibited a statistically significantly positive correlation (r = 0.937, *p* < 0.001 and r = 0.915, *p* < 0.001, respectively). A similar trend was observed in brachycephalic infants. Given the small sample size, respective correlation of HC and CV was not statistically assessed. In preterm infants, weight at TEA showed a statistically significant positive correlation with CV (r = 0.717, *p* < 0.001). In contrast, in term infants, only a trend but no significant correlation of body weight and CV (r = 0.359, *p* < 0.078) was found.

### Cranial proportion and (a)symmetry

**Figure 3:**
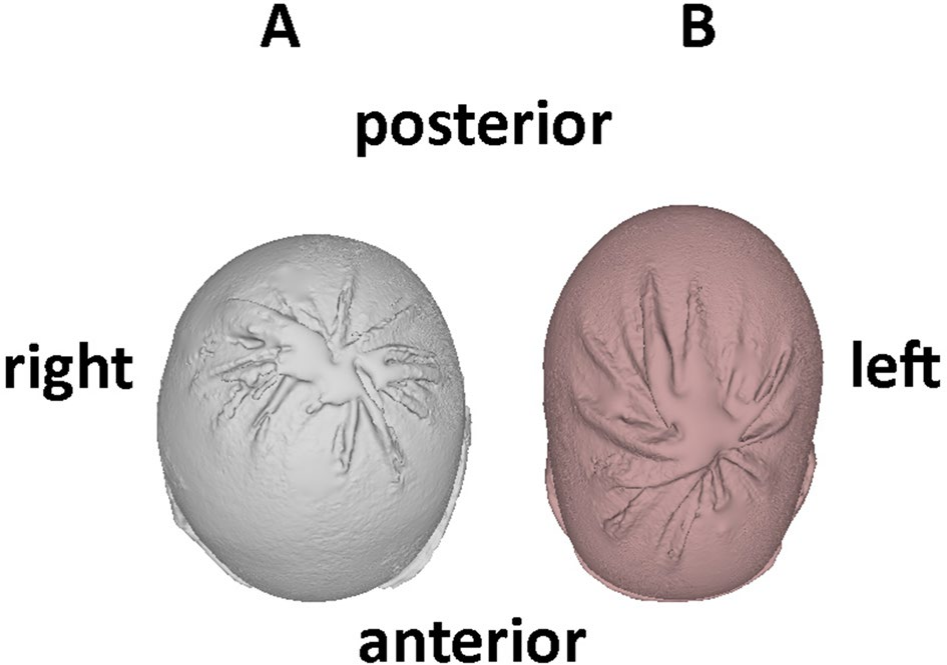
Transversal view of representative head shapes based on median head shape parameters of **term infants (A)** exhibiting a cranial index of 84.3% and a cranial volume of 735 cm^3^ compared to **preterm infants (B)** with a cranial index of 69.6% and a cranial volume of 738.7 cm^3^.

Preterm infants exhibited a higher degree of symmetrical head shape deformity (dolichocephaly) as reflected in a highly significantly lower CI when compared to controls (76% vs. 84%, p < 0.001). In contrast, CV was not significantly different between the two groups (Table 3). Brachycephaly was uncommon in term infants and did not occur in preterm infants. Similarly, we observed no significant differences for parameters representing head shape asymmetry (plagiocephaly). Representative head shapes of preterm and term infants are presented in Fig. 3. Preterms featuring lower CI at comparable head volumes is evident.

**Table 3:** Metric and volumetric evaluation of preterm and term infants ‘ heads.

	Preterm infants	Term infants	*P*
HC (cm)	35.1 (2.6)	34.5 (2.0)	0.041
CV (cm^3^)	688 (155)	648 (93)	0.059
CI (%)	76.0 (5.8)	84.3 (3.7)	< 0.001
CVAI (%)	3.1 (4.4)	2.5 (4.4)	0.496

*HC* head circumference; *CI* cranial index; *CV* cranial volume, *CVAI* cranial vault asymmetry index. Data are displayed as median (and interquartile range).

Occurrence and severity of specific head shape deformities at TEA are displayed in Table 4. There was a statistically significant association between preterm and term infants and the symmetry parameter CI (*p* = 0.006). This relation was determined as moderate (Cramer V = 0.432). Dolichocephaly was the most present head shape deformity in preterm infants. Specifically, dolichocephaly was mild in 8 / 25 (32%), moderate in 4 / 25 (16%), and severe in 5 / 25 preterm infants (20%), respectively.

**Table 4:** Symmetrical and asymmetrical head shape deformities in preterm and term infants.

		Preterm infants	Term infants
**Symmetrical**			
Brachycephaly	Moderate/severe (%)	0	8
	None/mild (%)	64	88
Dolichocephaly	Moderate/severe (%)	36	4
**Asymmetrical**			
Plagiocephaly	None/mild (%)	72	76
	Moderate/severe (%)	28	24

### Risk factors associated with head shape deformities

To assess potential risk factors for head shape deformities, all preterm and term infants were analysed regarding the presence of common clinical characteristics and risk factors including sex, multiple birth, abnormal birth presentation other than cephalic, mode of delivery and body weight at TEA. These parameters were not associated with brachycephaly, dolichocephaly or plagiocephaly.

Dolichocephaly was detected predominantly in preterm infants (36% vs. 4% in term infants). Within the preterm infant group, GA was significantly different between patients with dolichocephaly and without dolichocephaly (*p* = 0.049). However, there was no significant correlation between CI and GA (r = 0.262, *p* = 0.206). Likewise, there was no correlation of the severity of dolichocephaly with other risk factors such as total duration of respiratory support, days of nasal continuous positive airway pressure, nasal high-flow therapy or invasive mechanical ventilation, respectively.

**Figure 4:**
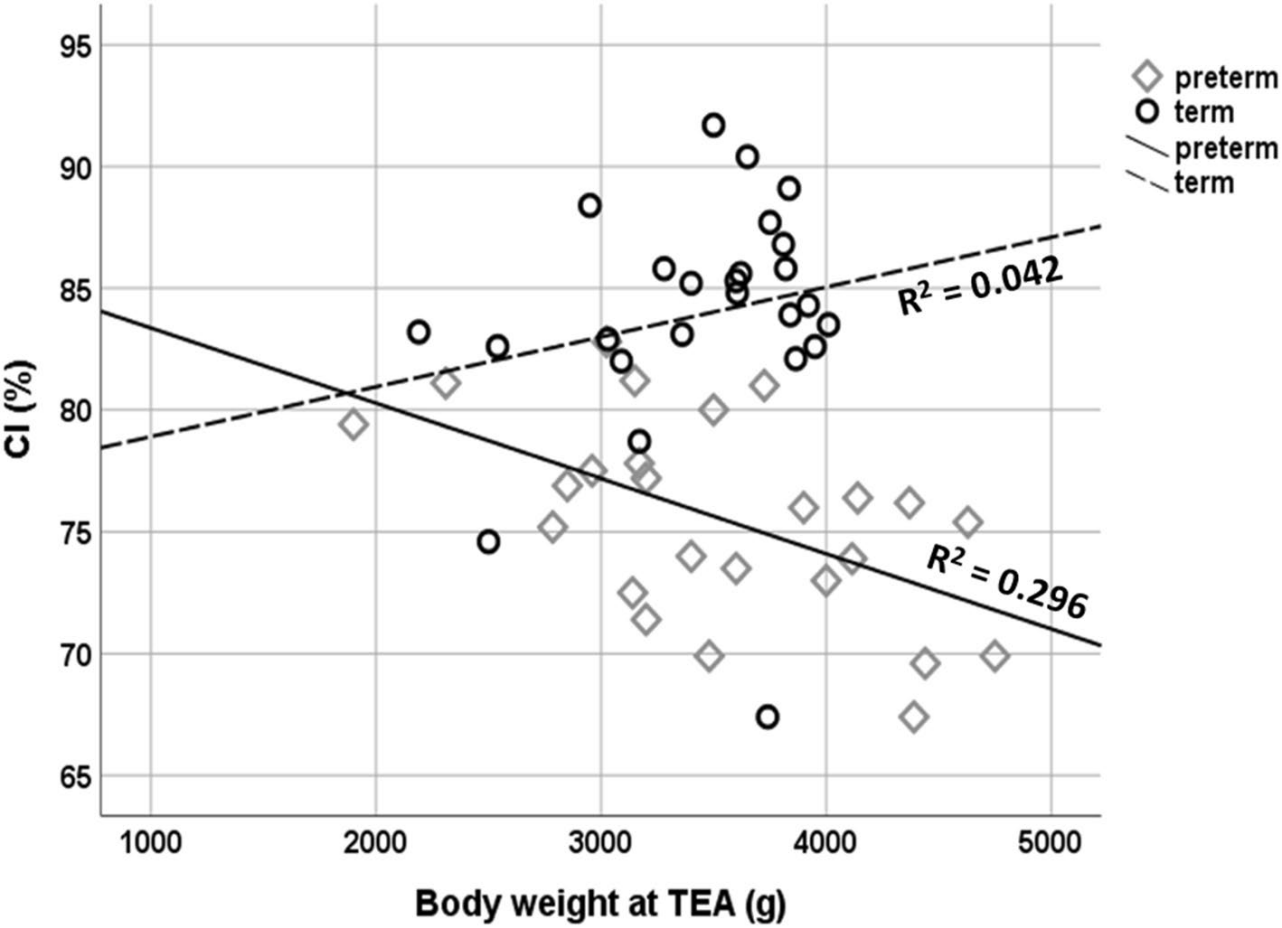
Correlation of body weight at term equivalent age and cranial index. *CI* cranial index, *TEA* term equivalent age.

To further investigate a potential effect of independent outcome variables on the CI in preterm infants, multiple hierarchical regression analysis was performed. Independent variables were sex, GA, presence of one or more criteria of neonatal morbidity (BPD, NEC, ICH III-IV°, sepsis, ROP III-IV°, PVL), total duration of respiratory support and body weight at TEA. On multiple hierarchical regression analysis, the only variable that accounted for a statistically significant effect on CI was body weight at TEA (Table 5***, ***Fig. 4). No significant effects were detected for any other of the above-mentioned variables.

**Table 5:** Results of the hierarchical multiple regression predicting cranial index.

		B	SE B	β	*P*
**Step 1** R^2^ = 0.296	Constant	86.449	3.571		** < 0.001**
	TEA	− 0.003	0.001	− 0.544	0.005

*B* unstandardized regression coefficients; *β* standardized regression coefficients; *SE* standard error; *TEA* term equivalent age.

## Discussion

Despite an increasing interest in the 3D head assessment with focus on non-synostotic head shape deformities, imaging of newborn heads still is only a small research field and reliable data remains limited. The main reasons for this shortcoming include technical limitations and the challenging need for a non-invasive, fast and accurate method that requires neither patient cooperation nor the use of sedatives in the newborn. Besides 2D methods, superior 3D techniques such as a laser shape digitizer have been described^20^ to address these issues. Yet to date, none of these techniques have been implemented into clinical routine. In this clinical study, a hand-held stereophotogrammetry device was successfully used in preterm and term infants at TEA allowing to create a database with the 3D parameters of the entire head. Metric and volumetric values as well as proportions and indices could be evaluated for all study participants.

Premature birth has been previously described as a risk factor for non-synostotic head deformities^21,22^. However, the exact circumstances determining the more frequent abnormal head shape development in preterm infants are unclear. Moreover, the timely evolution of head shape abnormalities in the first weeks to months after birth remains elusive.

To overcome this gap, here, we identified differences between preterm and term infants and aimed at characterizing general risk factors for head shape deformities for our entire patient cohort as well as specific risk factors for preterm infants. This is in accordance with results of Ifflaender et al., who used a laser shape digitizer to obtain 3D head images of preterm infants at TEA and who were able to determine differences when compared to term infants^19,20^. Unlike Ifflaender and colleagues, however, who found a significant difference in median duration of total respiratory support, nasal continuous positive airway pressure (nCPAP) and invasive mechanical ventilation in dolichocephalic infants compared to controls, we identified body weight at TEA to be an independent outcome variable with a potential effect on the CI.

### Relationship between head circumference, cranial volume and body weight

Early head and weight growth have been found to be associated with improved neurodevelopment in mid-childhood^23^. In the neonatal period, HC is—despite being only a 2D parameter—the current gold standard parameter for CV, and indirectly reflecting brain volume^9^. Accordingly, HC is considered a predictor for neurological outcome in neonates^13^.

In the present study, we analysed both HC and CV to determine whether the easy manual measurement of HC is equally applicable as the digital measurement of CV. In our patient cohort we found a high correlation between HC and CV. CV is estimated to be more comprehensive and therefore likely to be superior. Our data, however, supports the current clinical practice, that manually measured HC is a good instrument to determine CV.

Our findings are in agreement with results from a magnetic resonance imaging study by Cheong and colleagues who assessed head growth in preterm infants at TEA. In this study, microcephaly correlated with reduces brain tissue volumes^9^. Interestingly, and contrary to our expectations, HC mirrored CV even in patients with symmetrical as well as asymmetrical head deformities. However, since in our study cohort only a few patients had a severe head shape deformity, a larger number of patients needs to be studied to strengthen this statement.

In preterm infants, a highly significant correlation of body weight and CV could be identified. In contrast, in term infants this tendency was also present but did not reach statistical significance. It may be presumed, that differing chronological age and time-dependent factors between preterm and term infants account for this discrepancy. Examples for time-dependent factors could be the overlap of cranial sutures which is common in the first days after birth or physiological postnatal weight loss which is usually up to 7–10% of body weight at birth within the first days of life. Therefore, especially in term infants, weight might not be a reliable parameter to affect HC or CV at TEA.

### Cranial proportion and (a)symmetry

Between preterm and term infants at TEA, no clinically relevant differences were found for HC and CV. Applying the paradigm that a normal increase in HC and CV reflects physiological neurological development until TEA, this is a rather reassuring observation. Importantly, our patient cohort did not feature a high neonatal morbidity which may have influenced the favorable outcome in this study.

In accordance with the results by Ifflaender et al.^19^ and Willis et al.^10^, a lower CI—indicating the displaced relationship between head length and width—was found to be a discriminant characteristic between premature and term infants at TEA. Presence of a narrow and so-called “preemie head” is impressive for parents and caregivers but its biological impact still requires further evaluation. Plagiocephaly played a subordinate role in our study.

### Risk factors associated with dolichocephaly in preterm infants

For various reasons, preterm infants are susceptible for developing dolichocephaly. Theories for the underlying pathogenesis include preterm birth leading to exposure to gravitational forces at a very early age. Further, the cranial bone structures are more impressionable and therefore at higher risk to result in a sustained deformation of the head; neck muscle tone is reduced but still needs to hold a relatively larger head in place; and during hospitalization the head is regularly placed in a lateral position even more often when the baby is critically ill.

Interestingly, our multiregression analysis demonstrated that weight at TEA accounted for almost a third of all variance found in CI. In other words, a greater weight at TEA was associated with a greater risk for dolichocephaly. One possible explanation for this observation might be, that—according to Newton's law of gravity with mass being directly proportional to gravitational forces—larger infants are exposed to greater gravitational forces with the result of increased head shape deformation. A detailed head shape description of the only three preterm infants with a birth weight < 1000 g and body weights at TEA at < 1st, 2nd and 17th weight percentiles support this hypothesis: Only one baby had moderate dolichocephaly (CI 72.5%), the two others were measured within the upper normal range (CI 79.4% and 81.1%, respectively). With an average CI of 77.7%, these three lightest preterm infants (mean birth weight 703 g; mean weight at TEA 2450 g; one patient was SGA, microcephalic and showed a failure to thrive, another patient showed failure to thrive and was microcephalic at term) were less dolichocephalic than the average preterm infant in our study group (median CI of 76.0%). This is rather surprising, as we expected an increase in severity of head shape deformities among the SGA—as well as in the failure to thrive-infants, who are generally known to carry a greater risk for neonatal morbidity.

In our study, GA differed significantly between patients with dolichocephaly and without dolichocephaly but surprisingly not with duration of respiratory support or any other factors of neonatal morbidity. Although reliable evidence is sparse to support this assumption, one would expect a correlation between dolichocephaly and duration of nasal continuous positive airway pressure (nCPAP) support in particular, because the head cap which holds the nCPAP system fits rather tight around the infant´s head and might potentiate head deformation. Our data could not support these assumptions, and moreover, we did not find any difference between nCPAP and other invasive or non-invasive ventilatory modes.

## Limitations

The principle limitation of this study was the relatively small sample size. The reason for this was the pilot character of the study. However, the size of the preterm infant cohort was potentially too small to observe significant effects. Further, we did not define a specific hypothesis (e.g. based on cranial index cut-off values) to be proven wrong or right. Hence, the here described differences cannot be regarded statistically significant but rather hypothesis generating and descriptive in nature.

For our study, infants with known risk factors for head shape deformities such as multiple birth or birth presentation were not excluded which may have biased the results. Potentially, due to the small number of participants we did not find a correlation between any known risk factor and the presence of head shape deformities. In addition, the cohort was relatively healthy and neonatal morbidity was low. These factors may explain rather normal measurements in terms of HC and CV. Despite this, a significant number of infants showed relevant head shape deformities. The hypotheses generated in this study should be used to design adequately powered trials in the future.

## Outlook

Instead of focussing on single measurements, it would be interesting to assess head shape and volume development over time in order to identify potential confounders at different stages during infants´ growth as spontaneous improvement of head shape deformities often occurs during infancy.
